# High-Dose Methotrexate-Induced Idiopathic Intracranial Hypertension in Infant Acute Lymphoblastic Leukemia

**DOI:** 10.3389/fphar.2020.00839

**Published:** 2020-06-17

**Authors:** Yazhi Zhang, Yining Qiu, Zhujun Wang, Ran Wang, Runming Jin, Louis Edward Hinkle, Xiaoyan Wu

**Affiliations:** ^1^ Department of Pediatrics, Union Hospital, Tongji Medical College, Huazhong University of Science and Technology, Wuhan, China; ^2^ Department of Nanomedicine, Houston Methodist Research Institute, Houston, TX, United States

**Keywords:** high-dose methotrexate, neurotoxicity, idiopathic intracranial hypertension, dexamethasone, acute lymphoblastic leukemia

## Abstract

A 7-month-old baby girl with acute lymphoblastic leukemia (ALL) presented with bulging anterior fontanelle after completing the first and second courses of high-dose methotrexate (HD-MTX) chemotherapy. Between courses, the infant recovered and was discharged. Prior to the third and fourth HD-MTX courses, the baby girl was administered infusions of dexamethasone, which prevented recurrence of neurological side effects observed after the first and second courses of HD-MTX. To our knowledge, this is the first reported case of HD-MTX-induced idiopathic intracranial hypertension in infants, and that prophylactic use of dexamethasone can be applied to prevent acute intracranial hypertension following HD-MTX infusion.

## Introduction

A 7-month-old female patient with B-cell acute lymphoblastic leukemia (ALL) developed recurring anterior fontanelle bulging after completing both of her first and second courses of high-dose methotrexate (HD-MTX) induction. She did not, however, show any other abnormal neurological examinations, abnormal laboratory investigations, or abnormal brain CT. She recovered completely with mannitol and dexamethasone treatment. It appears that she benefited from prophylactic dexamethasone since she did not experience neurotoxicity following her third and fourth HD-MTX courses. Our clinical team concluded that the baby girl likely experienced idiopathic intracranial hypertension (IIH) induced by HD-MTX, which is characterized by increased intracranial pressure in the absence of any intracranial space-occupying lesion. Interestingly, we found that prophylactic use of dexamethasone could prevent foreseeable side effects of HD-MTX infusion, which has not been previously reported.

## Background

Methotrexate (MTX) is one of the most effective and widely used medications for the treatment and targeting of extramedullary leukemia (Pui et al., 2004). Although MTX is essential to pediatric ALL therapy, MTX therapy-induced toxicity remains a concern. MTX can cause multiple side effects including myelosuppression, hepatotoxicity, and neurotoxicity. MTX administration causes central nervous system neurotoxicity in 3.8 to 7.8% of ALL patients and manifests with transient stroke-like phenomena, which include altered mental status, seizures or seizure-like activity, and/or aphasia (Mahoney et al., 1998; Kishi et al., 2003; Bhojwani et al., 2014). Clinical symptoms of MTX-induced neurotoxicity are often associated with leukoencephalopathy, while the mechanisms underlying the pathogenesis are not fully understood. Acute intracranial hypertension is noticed as a low incidence and unpredictable consequence of MTX, occurring rarely in pediatric ALL patients (Inaba et al., 2007; Thon and Gittinger, 2016). Although MTX-related clinical neurotoxicity is transient, all symptomatic patients and one in five asymptomatic patients develop leukoencephalopathy. It is considered that prompt diagnosis and prevention of these side effects are crucial and that the outcome is favorable if the neurotoxicity is detected early (Weid and Popovic, 2006; Bhojwani et al., 2014).

In this case, we noticed that an infant presented idiopathic intracranial hypertension after receiving HD-MTX infusion, and we also found that dexamethasone might be used to prevent neurotoxicity in infants.

## Case

A 7-month-old female infant was diagnosed with B-cell ALL with an initially high WBC count. This baby girl was stratified into a moderate-risk group based on complicated findings and received the relevant courses of chemotherapy according to Chinese Children's Cancer Group ALL 2015 (CCCG-ALL-2015) protocol (Cai et al., 2019). This baby girl received timely induction therapies including Dexamethasone-preliminary induction, a VDLP course consisting of vindesine (VDS), daunorubicin (DNR), Pegasparagase (PEG-Asp), and prednisone, and the following CAM course of cyclophosphamide (CTX), cytarabine (Ara-C), and mercaptopurine (6-MP) with intrathecal therapy (I.T.). For this baby girl, there was no evidence of central nervous system (CNS) infiltration, and early assessment of minimal residual disease was negative. Genotype analysis for Methylenetetrahydrofolate reductase (MTHFR) variant C677T revealed high risk of MTX toxicity in this patient (Vagace et al., 2011).

Subsequently, this baby girl started a total of four courses of HD-MTX, with each course given every two weeks according to the CCCG-ALL-2015 protocol. The infusion in each course of HD-MTX lasted for 24 h. The first dose of HD-MTX was reduced to 3.5 g/m^2^ because of the patient's low creatinine clearance rate (CrCl). Forty-four hours after the start of MTX infusion, MTX serum level was 1.29 µmol/l and renal function was normal. Three days later, she presented symptoms of acute intracranial hypertension of tense and bulging anterior fontanelle and frequent vomiting. The baby girl also had a fever with 100 F (37.8°C) with normal neutrophil count. She was conscious and did not show discomfort from a headache. Blood pressure was normal and neurological examination was unremarkable. In laboratory investigation, the cerebrospinal fluid (CSF) showed no pleocytosis with normal protein and glucose concentrations, and brain CT was normal. The ophthalmologic exam and cranial MRI failed several times because she was too young to cooperate. The patient was given mannitol (1g/kg, 3 times daily IV) as soon as she developed symptoms and dexamethasone (0.25 mg/kg/d, 2 times daily IV) three days later because of persistent fontanelle bulging and vomiting. Her symptoms improved one day after dexamethasone treatment. Three days later she completely recovered, and we gradually reduced mannitol and dexamethasone over one week.Two weeks after the first MTX infusion, this infant patient received the second course of HD-MTX, which was decreased to 2.8g/m^2^. Forty-four hours after infusion, MTX serum level reached 6.60 µmol/l with normal renal function. Interestingly, she showed the same symptoms with high intracranial pressure and low-grade fever three days after completing the second course of HD-MTX chemotherapy. There was no evidence of intracranial infection or damage at this time. She received mannitol on the first day after symptoms and dexamethasone on the second day, with both treatments at the same dosages as before. After three days of treatment, the baby recovered completely and medications were gradually reduced.

**Figure 1:**
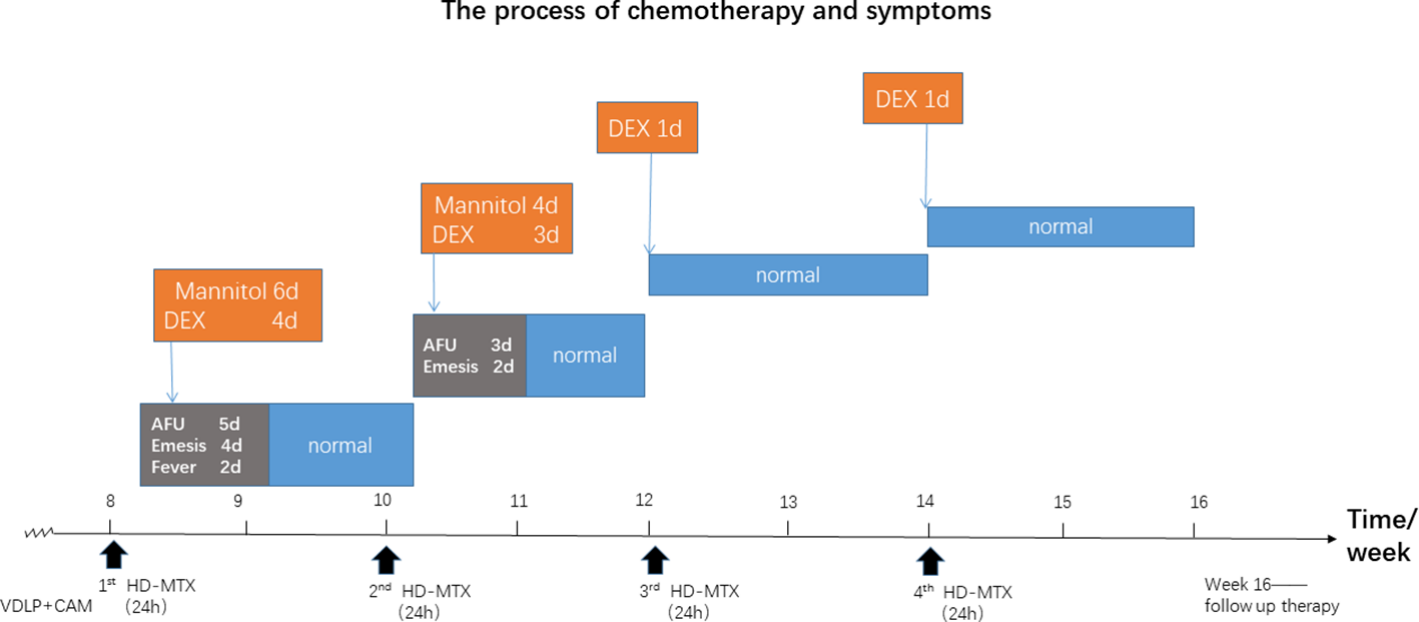
The chemotherapy process of the patient based on the Chinese Children's Cancer Group ALL 2015 (CCCG-2015-ALL) protocol. The red rectangle represents the symptoms after high-dose maintenance(HD-MTX) induction; The orange describes dexamethasone(DEX) and the other drugs therapy when symptoms where noticed. The blue refers to performances after therapy. AFU is short for anterior fontanelle uplift. The red triangle indicates the start of HD- MTX treatment.

Our clinical team considered that this infant's the disorder might have resulted from HD-MTX because she developed the same symptoms at the same point in time after each infusion of HD-MTX. Additionally, glucocorticoids like dexamethasone were found to be effective in quelling these side effects. Prior to the third and fourth infusion of HD-MTX, she was given the dexamethasone in advance to prevent intracranial hypertension, and, indeed, she did not present the same symptoms after the remaining infusions of HD-MTX. The process of chemotherapy is shown in **Figure 1**.

## Discussion

Here, we describe the case of an infant with elevated intracranial pressure at similar points in time after the first and second courses of HD-MTX infusion. MTX is an antimetabolite chemotherapeutic agent that is given intravenously, orally, and intrathecally. It has been a vital component of pediatric ALL therapy strategy for over 70 years, but it has the potential to cause significant clinical neurotoxicity and asymptomatic leukoencephalopathy, particularly when delivered intravenously (Ko and Liu, 2010). Side effect symptoms can include stroke-like phenomena, altered mental status, and seizures or seizure-like activity that occur up to 2 weeks after MTX exposure. However, acute intracranial hypertension is an unexpected adverse drug reaction for our infant patient since this side effect has mostly been described in adults cases (Phillips and Sheldon, 2017).

In the above infant patient, bulging anterior fontanelle presented after MD-MTX infusion without an identified etiology, such as an intracranial lesion, central nervous system infection, or other causes of elevated intracranial pressure. We examined and managed her condition with the suspicion that she had developed idiopathic IIH, which is a rare neurological disorder characterized by increased intracranial pressure in the absence of any intracranial space-occupying lesion. IIH, previously known as pseudotumor cerebri, may be a primary or secondary consequence to certain conditions (Warner et al., 2002). Over the past few decades, several medications have been described to be associated with IIH. There have been only a few reports of IIH associated with MTX in ALL children (Phillips and Sheldon, 2017). To our knowledge, this is the first case of MTX-induced IIH in infants.

A genetic predisposition may also play a role in susceptibility to the development of neurotoxicity following HD-MTX. Polymorphisms that contribute to MTX sensitivity have been identified in GSTP1, MTHFR, and SHMT1. Additionally, a recent genome-wide study has identified single nucleotide polymorphisms in genes involved in neuronal development pathways that contribute to MTX sensitivity (Bhojwani et al., 2014). The infant in our case was confirmed to have MTHFR (C677T) gene variant, and this potentially contributed to her MTX sensitivity. MTX Drug dosage was reduced according to her CrCl for the first infusion, and MTX dose was further decreased for the second infusion based on her 44 h MTX serum level, but she still experienced neurotoxicity.

The pathophysiology of IIH is incompletely characterized. Recent trials suggested that underpinning mechanisms of IIH include cerebrospinal fluid dysregulation as well as metabolic and endocrinological factors (Mollan et al., 2016). We hypothesize that HD-MTX might induce intracranial cell edema leading to increased intracranial pressure. Alternatively, HD-MTX could result in altered cerebrospinal fluid flow dynamics or hormone changes with ALL children (Donaldson, 1981; Burkett and Ailani, 2018). In the case presented here, the 7-month-old infant with intracranial hypertension showed recurring anterior fontanelle bulging because of her unclosed anterior fontanelle, which might help early detection and timely treatment of acute intracranial hypertension in infants.

It should be noted that dexamethasone might be an effective tool to treat and prevent such adverse reactions to MTX. We found that prophylactic dexamethasone was able to prevent IIH symptoms that would otherwise consistently appear in the infant patient following HD-MTX. This is a surprising finding when compared to previous research that showed corticosteroids used for treatment of autoimmune diseases like JIA and Crohn's disease could cause IIH after prolonged use in children and adults (Friedman et al., 2014; Kwon et al., 2016). There is a paucity of pediatric data to guide the use of medications in IIH, so there is much debate surrounding treatment of IIH in children. While in adults, acetazolamide was found to yield better outcomes and quality of life in the treatment of IIH (Bruce et al., 2016; Mollan et al., 2016). In the case we describe here, mannitol and dexamethasone were applied to the infant, and it was found that prophylactic dexamethasone was able to prevent IIH in the third and fourth HD-MTX infusions. Prophylactic use of dexamethasone to mitigate or prevent IIH is a novel concept and needs further investigation.In summary, since HD-MTX is a common drug for pediatric ALL patients to prevent extramedullary leukemia, caretakers of infant patients treated with HD-MTX should be warned against potential adverse effects, and prophylactic use of dexamethasone could prevent foreseeable acute intracranial hypertension resulting from HD-MTX infusion.

## Data Availability Statement

All datasets generated for this study are included in the article/supplementary material.

## Ethics Statement

Written informed consent was obtained from the parents of this patient for the publication of this case report and accompanying images. The Medical Ethics Committee of Union Hospital affiliated with Tongji Medical College of Huazhong University of Science and Technology approved this study, and the institutional review board gave us the permission to publish this case report and accompanying images.

## Author Contributions

YZ and YQ contributed to our collection of this patient's medical history. YZ and XW wrote the manuscript. XW approved of conceiving the report and edited the manuscript. LH helped to correct and improve the manuscript. RW and ZW collected the information during the follow-up period. RJ agreed to final approval of the version to be published. All authors reviewed and approved the manuscript.

## Conflict of Interest

The authors declare that the research was conducted in the absence of any commercial or financial relationships that could be construed as a potential conflict of interest.
